# Analysis of the expression and potential molecular mechanism of interleukin-1 receptor antagonist (IL1RN) in papillary thyroid cancer via bioinformatics methods

**DOI:** 10.1186/s12885-020-07620-8

**Published:** 2020-11-25

**Authors:** Zhenyu Xie, Xin Li, Yuzhen He, Song Wu, Shiyue Wang, Jianjian Sun, Yuchen He, Yu Lun, Shijie Xin, Jian Zhang

**Affiliations:** Department of Vascular and Thyroid Surgery, The First Hospital, China Medical University, Shenyang, China

**Keywords:** IL1RN, Papillary thyroid carcinoma, Diagnostic biomarker, Prognostic biomarker, Cancer immunity, IL-1

## Abstract

**Background:**

Interleukin-1 receptor antagonist (IL1RN) has been reported as a biomarker of many cancers. However, the biological function of IL1RN in papillary thyroid carcinoma (PTC) remains undetermined.

**Methods:**

We obtained IL1RN expression data from The Cancer Genome Atlas (TCGA) database. Enrichment analysis of coexpressed genes and IL1RN methylation analysis were performed via LinkedOmics. The correlations between IL1RN and immune infiltrates were investigated via ESTIMATE, TIMER and TISIDB. We analyzed the association of IL1RN expression with pancancer overall survival (OS) via Gene Expression Profiling Interactive Analysis (GEPIA).

**Results:**

IL1RN showed higher expression levels and lower methylation levels in PTC tissues than in normal tissues. Higher IL1RN expression was significantly associated with shorter progression-free survival (PFS), advanced tumor stage, tumor metastasis, increased incidence of BRAF mutations, and decreased incidence of N-RAS and H-RAS mutations. Genes coexpressed with IL1RN participate primarily in immune-related pathways. IL1RN expression positively correlated with immune infiltration, tumor progression and poor OS for all cancers.

**Conclusions:**

IL1RN is a good prognostic and diagnostic biomarker for PTC. IL1RN may promote thyroid cancer progression through immune-related pathways. Methylation may act as an upstream regulator of IL1RN expression and biological function. Additionally, IL1RN was shown to have broad prognostic value in a pancancer cohort.

**Supplementary Information:**

The online version contains supplementary material available at 10.1186/s12885-020-07620-8.

## Background

Thyroid carcinoma (THCA) is the most common endocrine malignancy worldwide [1]. The incidence of THCA has increased sharply over the past 3 decades [2]. Papillary thyroid carcinoma (PTC) is the major subtype of THCA, accounting for more than 90% of cases [3]. The clinical course of PTC is generally indolent, and the cancer-specific mortality of PTC is low compared to that of other cancers [4]. However, the incidence of cervical lymph node metastasis is high, which leads to local recurrence and poor prognosis [5]. The main task for the future is to identify high-risk patients and to give them appropriate treatment and care [6]. Therefore, further studies are required to explore the underlying mechanisms of tumorigenesis as well as identify additional biomarkers that predict prognosis and serve as therapeutic targets.

The interleukin-1 (IL-1) family of cytokines are the most effective molecules in the innate immune system [7]. IL-1 receptor antagonist (IL1RN) was initially found as a natural antagonist of IL-1 [8]. IL1RN is structurally related to IL-lα and IL-lβ but binds to IL-1 receptors on various target cells without inducing any discernible biological responses [9]. The balance between IL1RN and IL-1 plays a crucial role in many diseases, including cancer [10]. IL1RN has been studied in several cancers, including prostate carcinoma [11, 12], cervical carcinoma [13], gastric carcinoma [14], bronchogenic carcinoma [15], endometrial cancer [16], lung cancer [17], ovarian cancer [18], oral malignancies [19], leukemia [20], and other cancers.

Two structural variants of IL1RN have been described: the soluble extracellular form (sIL-1ra) and the intracellular form (icIL-1ra) [8]. Niedzwiecki, S. and colleagues assayed the serum levels of IL1RN in thyroid cancer patients. They measured preoperative IL1RN serum levels of patients with thyroid cancer, and the results showed that the serum concentrations of sIL-1ra were significantly higher in anaplastic carcinoma (ATC) and follicular carcinoma (FTC) patients [21]. To our knowledge, no reports have been published to date concerning IL1RN expression in thyroid tissue. Because IL1RN has been associated with various diseases and the serum concentration of sIL-1ra has been confirmed to be increased in PTC, we hypothesized that IL1RN may play a role in PTC. The objective of this study was to investigate IL1RN expression in normal and PTC tissues by performing bioinformatics analysis to elucidate its possible role in tumor progression.

## Methods

### Expression level analysis

The cohort that comprised 512 PTC and 58 normal thyroid samples was obtained from The Cancer Genome Atlas (TCGA) (https://tcga-data.nci.nih.gov/tcga/). The clinical data, normalized RNA expression data, DNA methylation data and simple nucleotide variation data were downloaded from the TCGA data portal.

To assess the diagnostic value of IL1RN, we selected data-sets containing both PTC and normal tissues in the GEO database. The gene expression profiles of four independent datasets (GSE3467, GSE33630, GSE58545, and GSE60542) were downloaded from the National Center for Biotechnology Information (NCBI) Gene Expression Omnibus (GEO) database (http://www.ncbi.nlm.nih.gov/geo/).

### Diagnostic and prognostic value analysis

ROC curves were plotted, and the area under the ROC curve was calculated using the ROCR package in R. The patients were divided into a high IL1RN expression group (H-IL1RN) and a low IL1RN expression group (L-IL1RN) according to the best matched value for the survival analysis. The best cut-off value was derived using Cutoff Finder software based on an R routine which optimized the significance of the split between Kaplan-Meier (K-M) survival curves measured by the log-rank test [22]. K-M survival curves were generated by the R survival package. The primary end point of the study was progression-free survival (PFS). Univariate and multivariate analyses were performed using the Cox regression model to assess the significance of various variables to survival. A chi-square test was performed to compare the clinical characteristics between the H-IL1RN group and the L-IL1RN group.

### Gene functional enrichment analysis

We identified the genes that were significantly positively or negatively correlated with IL1RN using the LinkedOmics website (http://www.linkedomics.org/) [23].

The top 50 positively correlated genes and the top 50 negatively correlated genes were selected to build the heatmaps.

These genes were input into the GO and KEGG websites to obtain the enriched GO terms and significant KEGG pathways. GO function annotation analysis was performed based on the GO database (http://geneontology.org/page/go-database), and KEGG pathway annotation analysis was performed based on the KEGG database (http://www.kegg.jp/kegg/ko.html).

The protein-protein interaction (PPI) network with a confidence > 0.7 was constructed using STRING (https://string-db.org) and CytoScape version 3.7.2.

### Tumor immunology analysis

ESTIMATE used the single-sample gene-set enrichment analysis (ssGSEA) score to quantify the enrichment levels of immune gene signatures in tumors. ESTIMATE [24], a method that uses gene expression signatures to infer the fractions of stromal and immune cells in tumor samples, was used to evaluate the levels of immune cell infiltration (immune score), the stromal content (stromal score), the stromal-immune comprehensive score (ESTIMATE score) and tumor purity for each THCA sample.

The Tumor Immune Estimation Resource (TIMER) web server (https://cistrome.shinyapps.io/timer/), a comprehensive analytic web tool [25], was used to analyze the correlation of IL1RN with infiltration of immune cells, including B cells, CD8+ T cells, CD4+ T cells, macrophages, neutrophils and dendritic cells (DCs).

We used TISIDB (http://cis.hku.hk/TISIDB/), a web portal for investigation of tumor and immune system interaction [26], to determine the Spearman correlation between IL1RN expression and 28 TIL types, chemokines, immune-activating cytokines, immunosuppressive cytokines, major histocompatibility complex (MHC) molecules, and chemokine receptors.

### Methylation-related analysis

DNA methylation data were downloaded from the data portal of TCGA (https://portal.gdc.cancer.gov/), and the DNA methylation levels of the PTC and control groups were then compared. Spearman correlation analysis was conducted to examine the associations between the methylation density and gene expression and between the methylation density and tumor purity. Additionally, we analyzed the correlation between IL1RN expression and the methylation level of each CpG site using the Spearman correlation test. The analysis of the relationship between methylation and the clinical characteristics and the GO and KEGG analyses were performed using linkedOmics.

### Pancancer analysis

The pancancer expression and survival analysis of IL1RN was performed using the online software Gene Expression Profiling Interactive Analysis (GEPIA) (http://gepia.cancer-pku.cn/) [27]. We used TISIDB to analyze the relationships between IL1RN expression and overall survival (OS) in pancancer. K-M curves were generated for pancancer using the TCGA cohort data.

### Related mutant gene analysis

The analysis of the relationship between IL1RN expression and PTC mutations was performed by linkedOmics. The IL1RN expression level was further compared between the groups with wild-type and mutant versions of BRAF, NRAS and HRAS.

### Statistical analysis

The *t*-test and Mann-Whitney U test were used for comparisons between two groups. The chi-square test was used to assess the differences in clinical parameters between the L-IL1RN and H-IL1RN groups. The Pearson and Spearman methods were used for correlation analysis. The log-rank method was used to calculate the significant *P*-values related to survival. R software (v3.6.0) and SPSS version 25.0 software were used for statistical processing. Visualization of data was performed with GraphPad Prism V.8.0 and R software. *P* < 0.05 was considered significant.

## Results

### The differential expression and diagnostic value of IL1RN

**Fig. 1:**
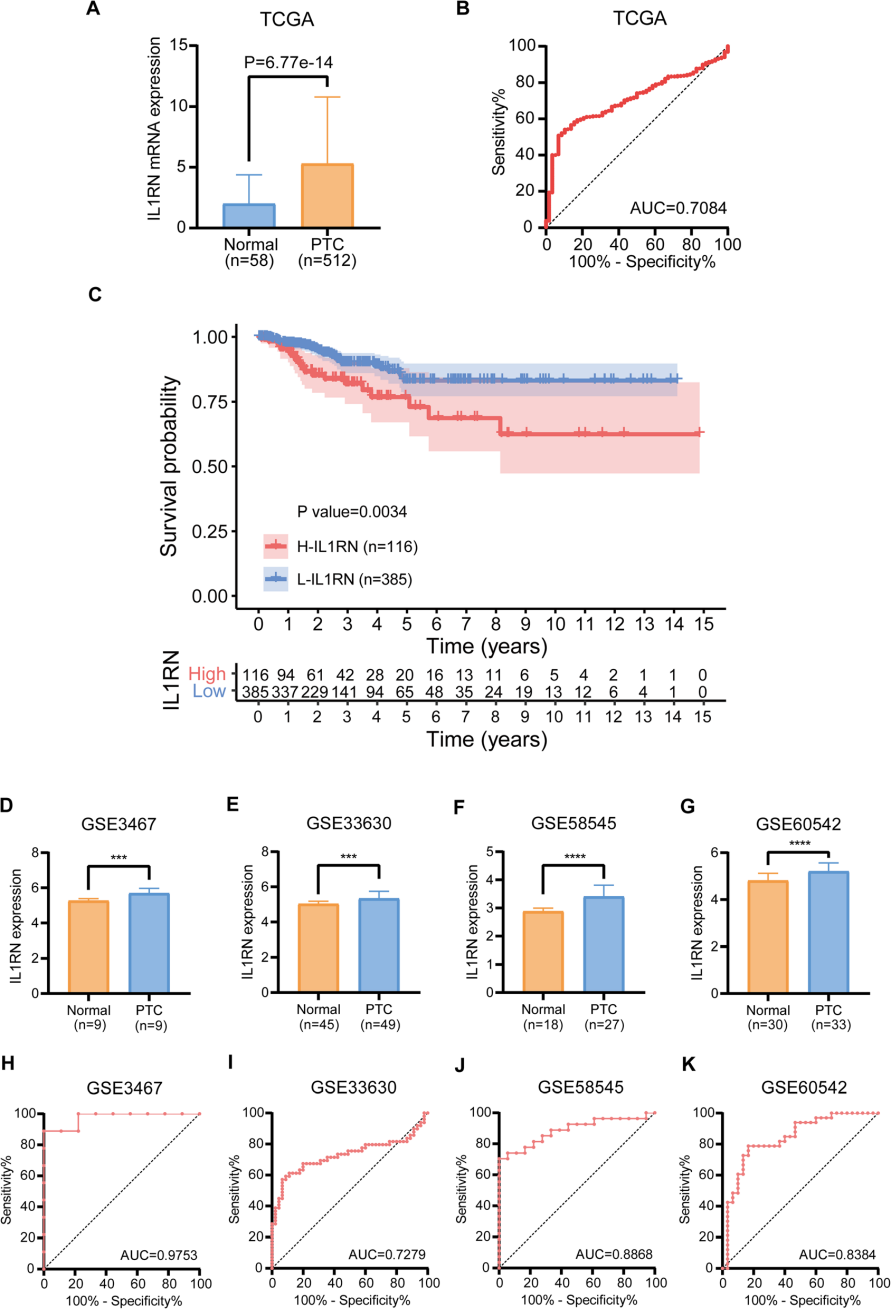
Diagnostic value of IL1RN in PTC. **a** Comparison of IL1RN mRNA expression between PTC and normal tissues in TCGA. **b** Diagnostic efficacy of IL1RN as shown by the ROC curve. **c** Kaplan–Meier curve for progression-free survival (PFS) in H-IL1RN group and L-IL1RN group. **d**-**g** mRNA expression of IL1RN in 4 GEO verification cohorts (GSE3467, GSE33630, GSE58545, GSE60542). (H-K) ROC curve showing the diagnostic performance of IL1RN expression in the verification cohorts. *P < 0.05, ** *P* < 0.01, *** *P* < 0.001, **** *P* < 0.0001 here and in the following figures

The expression levels of IL1RN were initially analyzed in the TCGA cohort consisting of 512 PTC samples and 58 normal samples (Fig. 1a). Furthermore, expression data from four independent PTC cohorts obtained from the Gene Expression Omnibus (GEO) (GSE3467, GSE33630, GSE58545, and GSE60542) were employed for validation (Fig. 1d-g). Both the GEO and TCGA patient cohorts had significantly higher IL1RN mRNA expression in PTC tissues than in normal tissues (*P* < 0.05).

ROC curves were constructed to evaluate the diagnostic value of IL1RN for PTC. The area under the ROC curve of the TCGA cohort was 0.7084 (Fig. 1b) and that of each GEO cohort was 0.9753, 0.7279, 0.8868, and 0.8384, respectively (Fig. 1h-k). These results suggest the good positive diagnostic value of IL1RN for PTC.

### Prognostic value and clinical significance of IL1RN

The PTC samples from the TCGA cohort were divided into two groups according to the IL1RN expression level: the high-expression group (H-IL1RN) (*n* = 116) and the low-expression group (L-IL1RN) (*n* = 385). Differences in progression-free survival (PFS) between the two groups were analyzed by Kaplan-Meier analysis (Fig. 1c). The results showed that patients with high IL1RN expression had significantly shorter PFS than those with low expression (*P* = 0.0034). However, the data for the multivariate analysis by the Cox regression model suggested that IL1RN expression was not a significant independent prognostic risk indicator for PTC (hazard ratio, 1.033; 95% confidence interval, 0.999–1.068, *P* > 0.05) (Table 1). Age was an independent predictor of poor prognosis (*P* < 0.05).

**Table 1:** Results of univariate and multivariate logistic regression analysis

Variables	Univariate analysis		Multivariate analysis	
	Hazard ratio (95%CI)	*P* value	Hazard ratio (95%CI)	*P* value
Age (y ≥ 55/< 55)	3.958 (2.274–6.891)	< 0.001	2.582(1.312–5.083)	0.006
Sex	1.471 (0.834–2.592)	0.182	1.098(0.604–1.997)	0.758
Clinical stage	1.736 (1.380–2.185)	< 0.001	1.301(0.968–1.748)	0.081
Radiation therapy	1.305 (0.728–2.343)	0.372	1.205(0.651–2.233)	0.553
IL1RN	1.058 (1.024–1.093)	< 0.001	1.033(0.999–1.068)	0.059

To examine the clinical significance of IL1RN in PTC, the relationship between the expression level of IL1RN and clinical characteristics was investigated by the chi-square test (Table 2). The results suggested that IL1RN expression was significantly associated with clinical stage, N stage, T stage, pathologic type, BRAF mutations and RAS mutations.

**Table 2:** Association between IL1RN expression and clinical parameters

Clinical parameters	L-IL1RN (*n* = 385, %)	H-IL1RN (*n* = 116, %)	P-value
**Age (y)**			
< 55	261 (67.8)	73 (62.9)	0.330
≥ 55	124 (32.2)	43 (37.1)	
**Sex**			
Female	282 (73.2)	84(72.4)	0.859
Male	103 (26.8)	32(27.6)	
**Clinical stage**			
I	224 (58.5)	57 (49.1)	0.001
II	47 (12.3)	5 (4.3)	
III	77 (20.1)	34 (29.3)	
IV	35 (9.1)	20 (17.2)	
NA	2	0	
**Metastasis**			
M0	208 (97.2)	74 (96.1)	0.635
M1	6 (2.8)	3 (3.9)	
NA	171	39	
**N classification**			
N0	189 (54.8)	40 (37.7)	0.002
N1	156 (45.2)	66 (62.3)	
NA	40	10	
**T classification**			
T1	120 (31.2)	22 (19.3)	< 0.001
T2	135 (35.1)	29 (25.4)	
T3	116 (30.1)	54 (47.4)	
T4	14 (3.6)	9 (7.9)	
NA	0	2	
**Pathologic type**			
Classical	268 (69.6)	87 (75.0)	< 0.001
Follicular	94 (24.4)	7 (6.0)	
Tall Cell	17 (4.4)	19 (16.4)	
Other	6 (1.6)	3 (2.6)	
**BRAF**			
Wild	179 (48.5)	16 (14.3)	< 0.001
Mutation	190 (51.5)	96 (85.7)	
NA	16	4	
**RAS**			
Wild	311 (84.3)	110 (98.2)	< 0.001
Mutation	58 (15.7)	2 (1.8)	
NA	16	4	

### Gene coexpression and pathway enrichment analysis

**Fig. 2:**
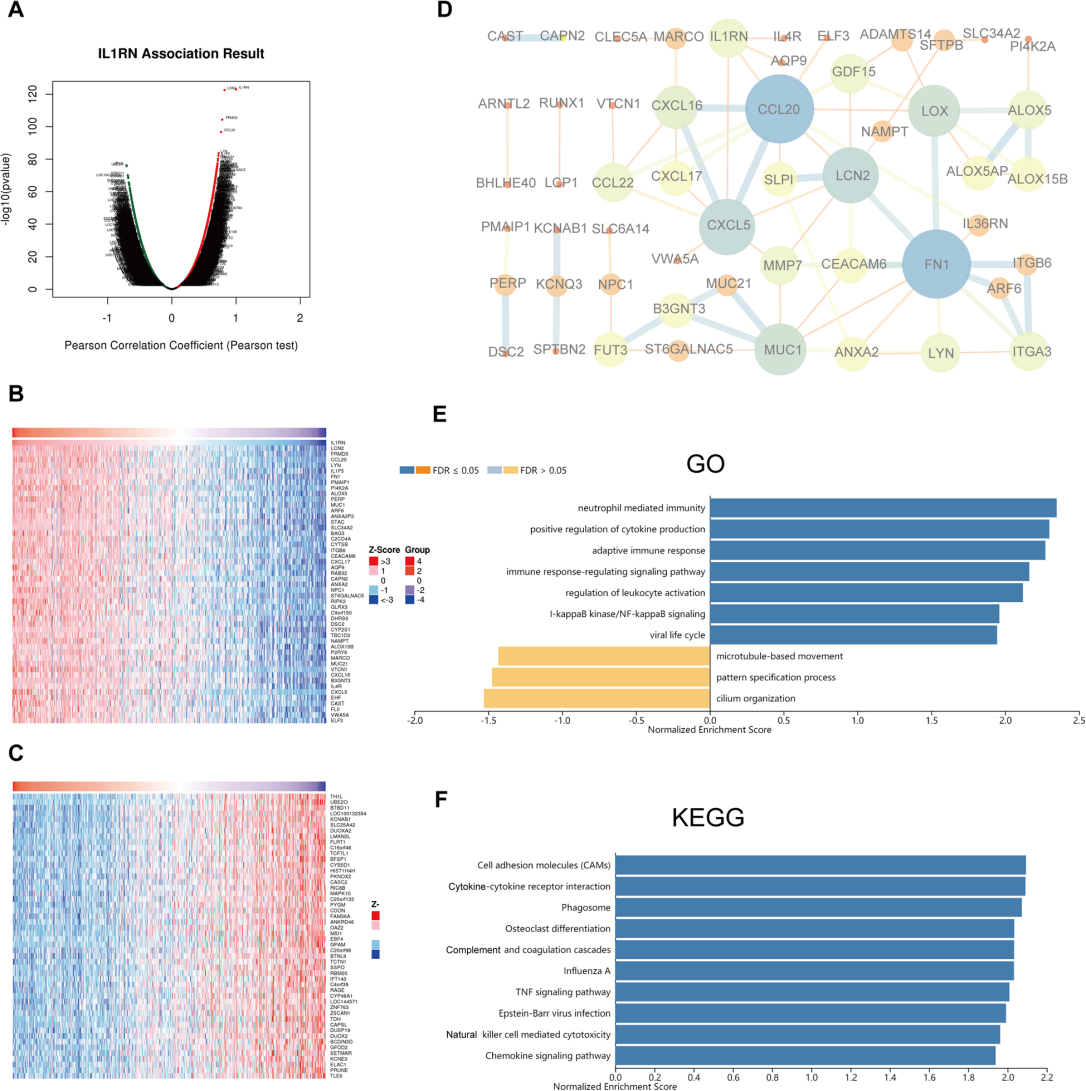
Potential mechanisms of IL1RN in PTC. **a** Genes highly correlated with IL1RN identified by Pearson correlation analysis in the THCA cohort. **b** Heatmaps showing the top 50 genes positively and (**c**) negatively correlated with IL1RN in the TCHA cohort. **d** PPI of the top 100 significantly correlated genes. **e** GO biological process terms and (**f**) KEGG pathways significantly enriched in genes coexpressed with IL1RN in the THCA cohort

The *Function* module of LinkedOmics was used to analyse mRNA sequencing data from 512 PTC patients in TCGA. The result is presented as a volcano plot (Fig. 2a). A total of 8960 genes showed significant positive correlations with IL1RN(red dots), while 10,967 genes showed significant negative correlations with IL1RN (green dots). The top 50 positively (Fig. 2b) or negatively (Fig. 2c) correlated genes are depicted by heatmaps. In addition, 2 hub genes (CCL20 and FN1) were identified from the protein–protein interaction (PPI) network (Fig. 2d).

We used gene set enrichment analysis (GSEA) to conduct GO term and KEGG analyses for all positive IL1RN co-expressed or negative IL1RN co-expressed genes. GO enrichment analysis revealed that IL1RN co-expressed genes were mainly enriched for the GO biological process terms “neutrophil mediated immunity”, “positive regulation of cytokine production”, “adaptive immune response” and “immune response-regulating signalling pathway” (Fig. 2e). Further KEGG enrichment analysis suggested that the identified genes were mainly involved in pathways associated with “cell adhesion molecules (CAMs)”, “cytokine-cytokine receptor interaction”, and “phagosome” (Fig. 2f).

### Immune-related analysis of IL1RN

**Fig. 3:**
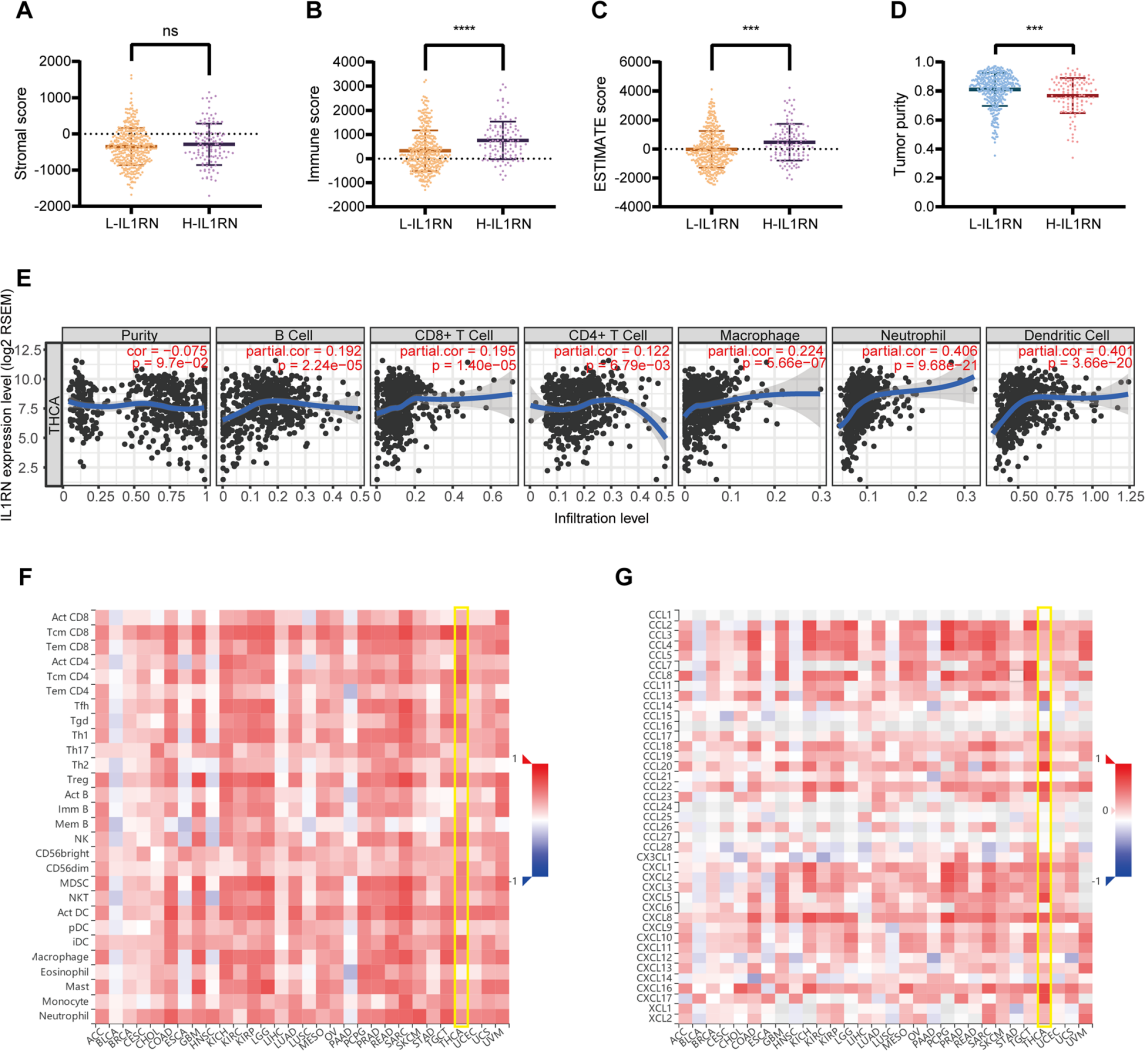
IL1RN is closely related to immunity in PTC. ESTIMATE analysis of (**a**) stromal scores, (**b**) immune scores, (**c**) ESTIMATE scores, and (**d**) tumor purity between the L-IL1RN and H-IL1RN groups. **e** TIMER analysis of purity-corrected partial Spearman correlations between the expression of IL1RN and the infiltration of six types of immune cells in the THCA cohort. **f** Correlation analysis between the expression of IL1RN and 28 types of TILs across human cancers via TISIDB. **g** Correlation analysis between the expression of IL1RN and the levels of chemokines across human cancers via TISIDB

Using ESTIMATE, the association between IL1RN expression and immune infiltrates was analyzed. We found that the H-IL1RN group showed a higher immune score (Fig. 3b) and ESTIMATE score (Fig. 3c) and lower tumor purity (Fig. 3d) than the L-IL1RN group. No significant difference in the stromal score was found between the two groups (Fig. 3a). The correlations between the IL1RN expression level and the infiltration of six immune cell types in PTC were estimated. The results demonstrated that IL1RN expression was found to be significantly positively correlated with the infiltration of immune cells, especially neutrophils and dendritic cells (Fig. 3e). Correlations between IL1RN expression and 28 TIL types (Fig. 3f), chemokines (Fig. 3g), immune-activating cytokines (Fig. S1A), immunosuppressive cytokines (Fig. S1B), MHC molecules (Fig. S1C), and chemokine receptors (Fig. S1D) in pancancer are shown in heatmaps. A positive correlation between IL1RN expression and immune-related molecules was observed.

### Methylation-related analysis

**Fig. 4:**
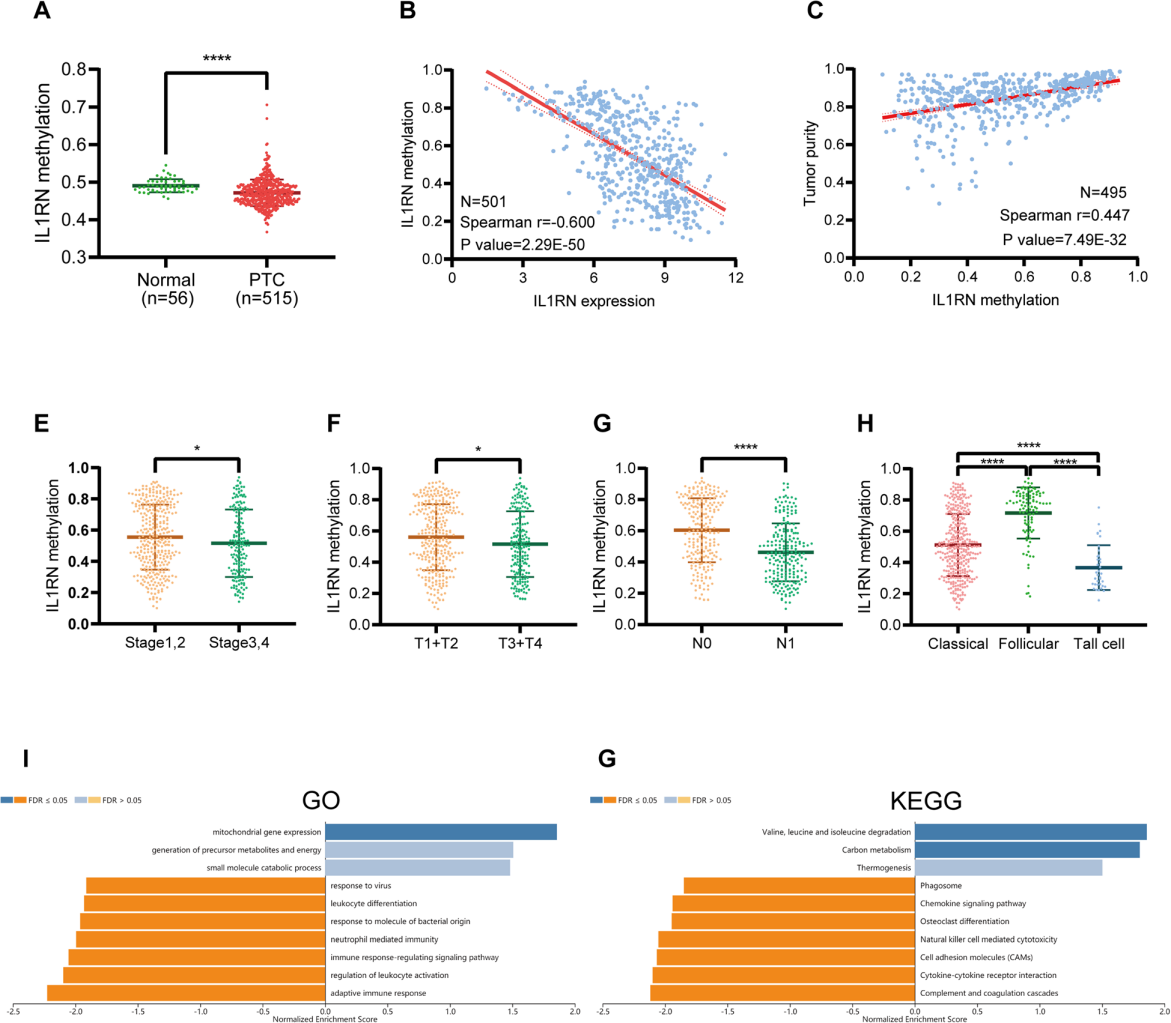
Methylation of IL1RN in PTC. **a** IL1RN methylation levels were compared between PTC and normal tissues. **b** Correlation analysis between IL1RN methylation and IL1RN expression in PTC. **c** Correlation analysis between IL1RN methylation and tumor purity in PTC. IL1RN methylation level among patients with different (**d**) clinical stages, (**e**) T classifications, (**f**) N classifications, and (**g**) histological types of PTC. **h** GO biological process terms and (I) KEGG pathways significantly altered by IL1RN methylation in the THCA cohort

The data showed that the degree of IL1RN DNA methylation was lower in carcinoma tissues than in normal tissues (*P* < 0.0001) (Fig. 4a). To further determine the role of methylation, we performed a correlation analysis between the DNA methylation levels of IL1RN and the expression levels of IL1RN. As expected, DNA methylation negatively correlated with IL1RN expression (R = -0.600, *P* = 2.29e-50) (Fig. 4b). Tumor purity was significantly positively associated with IL1RN methylation (R = 0.447, *P* = 7.49e-32) (Fig. 4c). Patients with an advanced clinical stage (Fig. 4d), advanced T stage (Fig. 4e) and lymph node metastasis (Fig. 4f) tended to have decreased IL1RN methylation levels. In the pathological subtype of PTC, the tall cell type variant of PTC had the lowest degree of methylation, followed by the classical type, and the follicular variant of PTC had the highest degree of methylation (Fig. 4g).

To understand the biological significance of IL1RN methylation in THCA, a functional module of LinkedOmics was used to examine IL1RN coexpression pattern in the THCA cohort. Based on RNAseq, we screened 19,927 genes related to IL1RN methylation (false discovery rate (FDR) < 0.01). The GO (biological process) analysis results derived by GSEA were significant. The results indicated that IL1RN methylation coexpressed genes that participate primarily in mitochondrial gene expression, the generation of precursor metabolites and energy and small molecule catabolic process, while immune related activities, such as the adaptive immune response, immune response-regulating signalling, and neutrophil mediated immunity were inhibited (Fig. 4h). KEGG pathway analysis also showed that genes related to valine, leucine and isoleucine degradation, carbon metabolism and thermogenesis, among other pathways, were inhibited (Fig. 4i). These results illustrate that methylated IL1RN may inhibit immune-related pathways by downregulating IL1RN expression. Furthermore, the most significant methylation sites are shown in Table 3.

**Table 3:** Spearman correlation between IL1RN methylation sites and IL1RN expression

Methylation site	Spearman r	*P* value
cg01467417	−0.597	7.76E-50
cg02543462	−0.406	2.53E-21
cg01991967	−0.19	1.81E-05
cg03989987	−0.164	2.28E-04
cg10938446	− 0.116	0.009
cg03703171	−0.107	0.016
cg11783497	− 0.1	0.016
cg17669033	0.027	0.548
cg23041410	−0.022	0.625
cg06658391	0.018	0.687
cg25928199	NA	NA
cg25265126	NA	NA
cg02377053	NA	NA

### Related mutant gene analysis

The analysis of the relationship between IL1RN expression and PTC mutations was performed by LinkedOmics (Fig. S2A). The IL1RN expression level was further compared between the groups with wild-type and mutant variants of BRAF, NRAS and HRAS (Fig. S2B-D). The expression of IL1RN was significantly higher in PTC with the BRAF mutant than in PTC with the wild type (Fig. S2B). However, mutated NRAS (Fig. S2C) and mutated HRAS (Fig. S2D) correlated with decreased IL1RN expression.

### Pancancer analysis of IL1RN

**Fig. 5:**
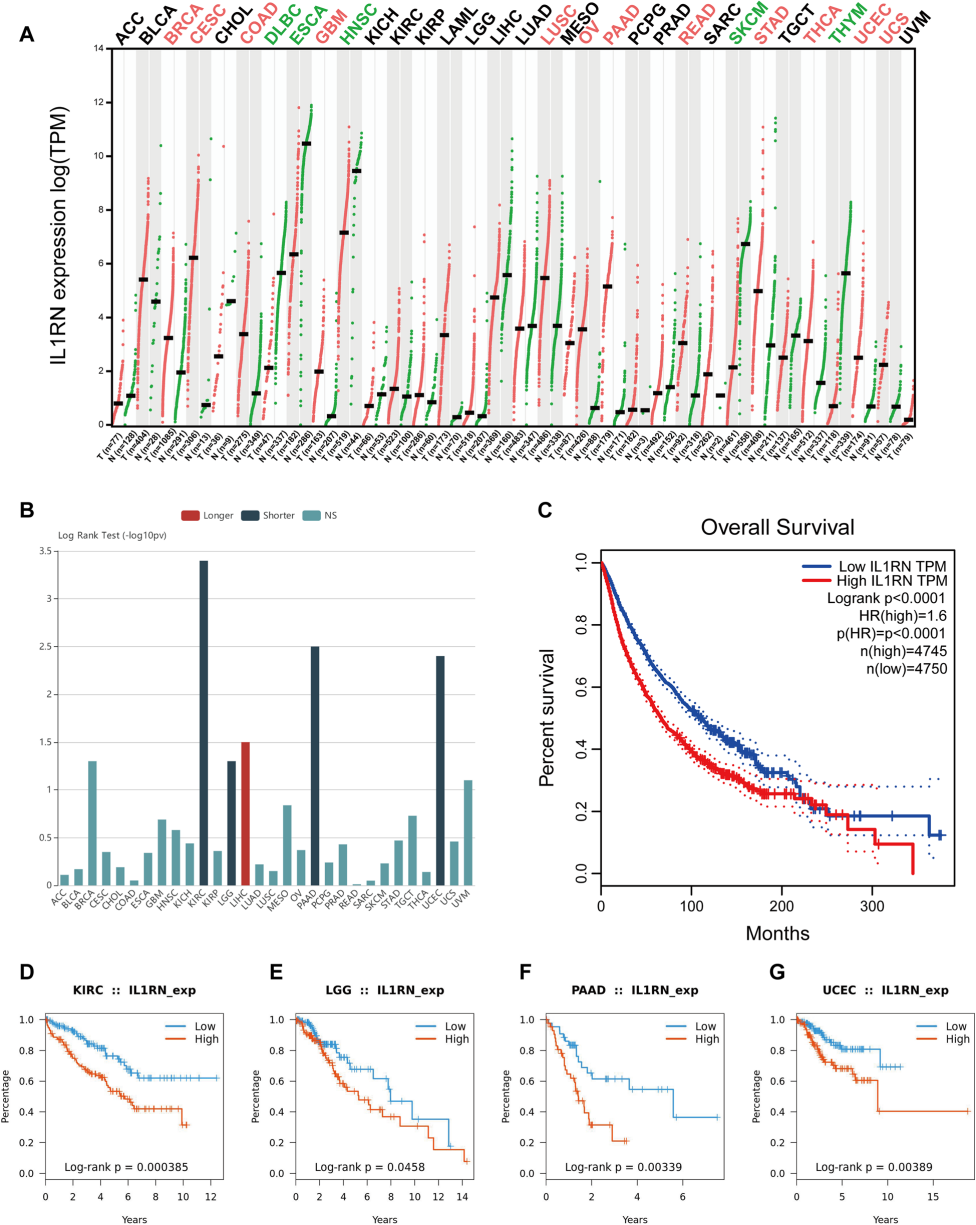
Generalization value of IL1RN across cancer. **a** Comparison of IL1RN mRNA expression between cancer and paracancerous tissuess across cancers. **b** Associations between IL1RN expression and OS across human cancers. K-M survival analysis between the L-IL1RN and H-IL1RN in (**c**) pan-cancer cohorts, **d** KIRC, **e** LGG, **f** PAAD, and **g** UCEC

To investigate whether IL1RN has broad value, we performed a series of studies on IL1RN across all cancers. We analyzed the IL1RN expression levels in different kinds of tumors via the GEPIA platform (Fig. 5a). The results showed that the expression levels of IL1RN varied greatly in different cancer types. Of the 33 cancer types tested, 12 cancer types were associated with significantly increased IL1RN expression. Subsequently, the relationship between IL1RN and OS in pancancer was investigated (Fig. 5b). Kaplan-Meier survival curves for the high IL1RN expression group (4745 patients) and low IL1RN expression group (4750 patients) indicated that increased IL1RN expression was associated with a shorter survival time in pancancer (HR = 1.6, *P* < 0.0001) (Fig. 5c). The results showed that increased IL1RN expression levels were significantly associated with shorter OS in kidney renal clear cell carcinoma (KIRC) (Fig. 5d), brain lower grade glioma (LGG) (Fig. 5e), pancreatic adenocarcinoma (PADD) (Fig. 5f) and uterine corpus endometrial carcinoma (UCEC) (Fig. 5g).

## Discussion

In this study, we first revealed higher IL1RN expression in PTC tissue compared than in normal tissues and its diagnostic and prognostic value by integrated bioinformatics analysis. To further explore the potential function of IL1RN, we identified genes related to IL1RN expression, and functional enrichment analyses were conducted. The results of the KEGG pathway and GO analyses revealed the significant enrichment of genes in immune-related pathways. Our work further demonstrated that high IL1RN expression was significantly associated with decreased tumor purity and increased immune infiltration. Through methylation-related analysis, we determined that DNA methylation might be a regulatory mechanism of IL1RN. In addition, the analysis of the relationship between IL1RN expression and PTC mutations showed that high IL1RN expression was associated with mutated BRAF, wild-type NRAS and wild-type HRAS. Finally, IL1RN shows superior prognostic value for various cancers according to the pancancer analysis.

Niedzwiecki et al. [21] revealed that the serum levels of IL1RN were upregulated in patients with ATC and PTC, but no statistically significant difference was found in PTC. We found that the expression of IL1RN was increased in PTC tissues compared with that in normal tissues in 1 TCGA cohort and 4 GEO cohorts and suggested that IL1RN might be used as a potential diagnostic biomarker in PTC. We guess that overexpression of IL1RN in thyroid tissues of PTC patients result in elevated levels of serum IL1RN. Thus, the findings of Niedzwiecki et al. corroborate our results. Our results were consistent with those of previous reports on the upregulation of IL1RN in cervical carcinoma [13], gastric cancer [14], lung cancer [15], and endometrial cancer tissues [16]. These findings are also basically consistent with the results of our pancancer study. In contrast to these findings, IL1RN exhibited low expression in oral squamous cell carcinomas (OSCCs) [19].

The results of this study showed the value of IL1RN as a clinical biomarker in PTC and emphasized its potential as a prognostic biomarker in PTC patients. PTC patients with high IL1RN expression had decreased PFS compared to those with low IL1RN expression. Furthermore, high expression of IL1RN was significantly correlated with clinical stage, lymph node metastasis and pathological type. Various studies have highlighted the significant association between IL1RN expression and poor cancer prognosis; however, the results of these studies are conflicting. Some studies revealed that there was a significant positive correlation between poor prognosis and the expression of IL1RN [14, 16, 28]. In contrast, low levels of IL1RN have been associated with increased disease severity in myeloma [29], colorectal cancer [30] and prostate cancer [31].

The underlying mechanism of IL1RN in cancer development and progression is complicated and unclear. In this study, functional enrichment analysis of genes coexpressed with IL1RN showed that IL1RN participates in immune-related biological processes. Consistent with our findings, previous studies have focused on the role of IL1RN in tumor immunity. A study of gastric carcinoma [14] pointed out that on the one hand, IL1RN may promote tumor growth via the impairment of cellular immunity; on the other hand, IL1RN enables malignant cells to escape host immune responses. Smith, D. R. et al. [15] revealed that increased IL1RN in bronchogenic carcinoma is not accompanied by increased IL-1β activity. The altered balance of IL1RN and IL-1β may result in impaired immune surveillance and cytotoxic activity. An experimental study of human glioblastoma cells showed that IL1RN secreted by tumor cells can counteract IL-1 function, which represents a potential escape mechanism that supports cancer growth [32]. In our study, IL1RN was significantly positively correlated with lymph node metastasis and tumor stage, so we speculated that IL1RN might also promote tumor aggressiveness and poor prognosis through immune-related mechanisms in PTC.

IL1RN is an endogenous natural antagonist of IL-1 [8], so the discovery of the interaction between IL1RN and IL-1 family molecules is a breakthrough in exploring the function of IL1RN. Onozaki et al. [33] reported that IL-1 is a cytocidal factor against several tumor cell lines. IL-1 may enhance cytotoxic T cell activity [34], the tumoricidal capacities of natural killer (NK) cells [35], and monocyte-mediated cytotoxicity [36]. Because IL-1 is critical for tumor immunity, elevation of IL1RN expression may result in a general environment favourable to tumor cells and enhance the metastatic and recurrence potential of tumors by changing local IL-1-dependent pathways.

IL-1β has been reported as an anticancer factor that acts to suppress proliferation and reduce the invasive potential of human PTC cells [37]. sIL-1ra has been shown to block IL-1 function by binding to IL-1 receptors at the cell membrane level [34]. IcIL-1ra is postulated to inhibit intracellular IL-1 activity [38]. Therefore, whether IL1RN can block the function of IL-1 and inhibit the anticancer effect of IL-1β in PTC is worth further study.

In our study, IL1RN showed a significant positive correlation with immune cells, which may be because a variety of immune cells can produce IL1RN through the stimulation of cytokines. Neutrophils produce IL1RN in response to granulocyte/macrophage colony stimulating factor (GM-CSF) and tumor necrosis factor-α (TNF-α) [39]. IL-4 and IL-10 have been reported to increase the production of IL1RN by human monocytes [36]. Yanagawa, H.et, al. reported that IL-13 increases IL1RN production by human alveolar macrophages [40]. It was reported that the apoptotic cell death of monocytes was enhanced by administration of recombinant IL1RN [41]. Our study also showed a significant correlation between IL1RN expression and various cytokines, which implied that IL1RN may play a complex role in tumor immunity.

Our study suggests that methylation is a pretranscriptional regulatory mechanism for IL1RN. The methylation level of IL1RN was negatively correlated with its expression level and was also correlated with the disease stage, lymph node metastasis, and pathological type.

IL1RN is likely to be a potential biomarker associated with the diagnosis and prognosis of PTC. However, although the diagnostic value, prognostic value and molecular functions of IL1RN in PTC have been analyzed through bioinformatics methods, the conclusions have not yet been confirmed by experiments. Therefore, further research is necessary to explore the role of IL1RN in PTC and the pharmacological value of IL1RN as a therapeutic target.

## Conclusions

IL1RN is a good prognostic and diagnostic biomarker of PTC. IL1RN may promote thyroid cancer progression through immune-related pathways. Methylation may act as an upstream regulator of IL1RN expression and biological function. Additionally, IL1RN showed broad prognostic value in an analysis of a pancancer cohort.

## Supplementary information

Additional file 1: **Fig. S1.** IL1RN expression is closely related to immunity across human cancers. Correlation analysis between the expression of IL1RN and the levels of (A) immune activating cytokines, (B) immune suppressive cytokines, (C) MHCs, and (D) chemokine receptors.

Additional file 2: **Fig. S2.** Relationship between IL1RN expression and gene mutations in PTC. (A) Volcano plot of IL1RN expression and gene mutations in THCA. Relationship between IL1RN expression and (B) BRAF mutation, (C) NRAS mutation, and (D) HRAS mutation.

## Data Availability

The datasets analysed during the current study are available in The Cancer Genome Atlas (TCGA) (https://tcga-data.nci.nih.gov/tcga/) or the National Center for Biotechnology Information (NCBI) Gene Expression Omnibus (GEO) database (http://www.ncbi.nlm.nih.gov/geo/). The raw data may be made available upon reasonable request from the corresponding authors.

## Abbreviations

IL1RN: Interleukin-1 receptor antagonist
PTC: Papillary thyroid carcinoma
TCGA: The Cancer Genome Atlas
OS: Overall survival
GEPIA: Gene Expression Profiling Interactive Analysis
PFS: Progression-free survival
THCA: Thyroid carcinoma
IL-1: Interleukin-1
sIL-1ra: The soluble extracellular form of IL1RN
icIL-1ra: The intracellular form of IL1RN
ATC: Anaplastic carcinoma
FTC: Follicular carcinoma
NCBI: The National Center for Biotechnology Information
GEO: Gene Expression Omnibus
H-IL1RN: High IL1RN expression group
L-IL1RN: Low IL1RN expression group
PPI: Protein-protein interaction
ssGSEA: Single-sample gene-set enrichment analysis
TIMER: Tumor Immune Estimation Resource
DCs: Dendritic cells
MHC: Major histocompatibility complex
K-M: Kaplan-Meier
CAMs: Cell adhesion molecules
KIRC: Kidney renal clear cell carcinoma
LGG: Lower grade glioma
PADD: Pancreatic adenocarcinoma
UCEC: Uterine corpus endometrial carcinoma
OSCCs: Oral squamous cell carcinomas
NK: Natural killer
GM-CSF: Granulocyte/macrophage colony stimulating factor
TNF-α: Tumor necrosis factor-α
