# Supplementary figures and images for: Analysis of the expression and potential molecular mechanism of interleukin-1 receptor antagonist (IL1RN) in papillary thyroid cancer via bioinformatics methods
Source: BMC Cancer. 2020 Nov 25;20:1143. doi: 10.1186/s12885-020-07620-8 (PMC7687764; doi:10.1186/s12885-020-07620-8)

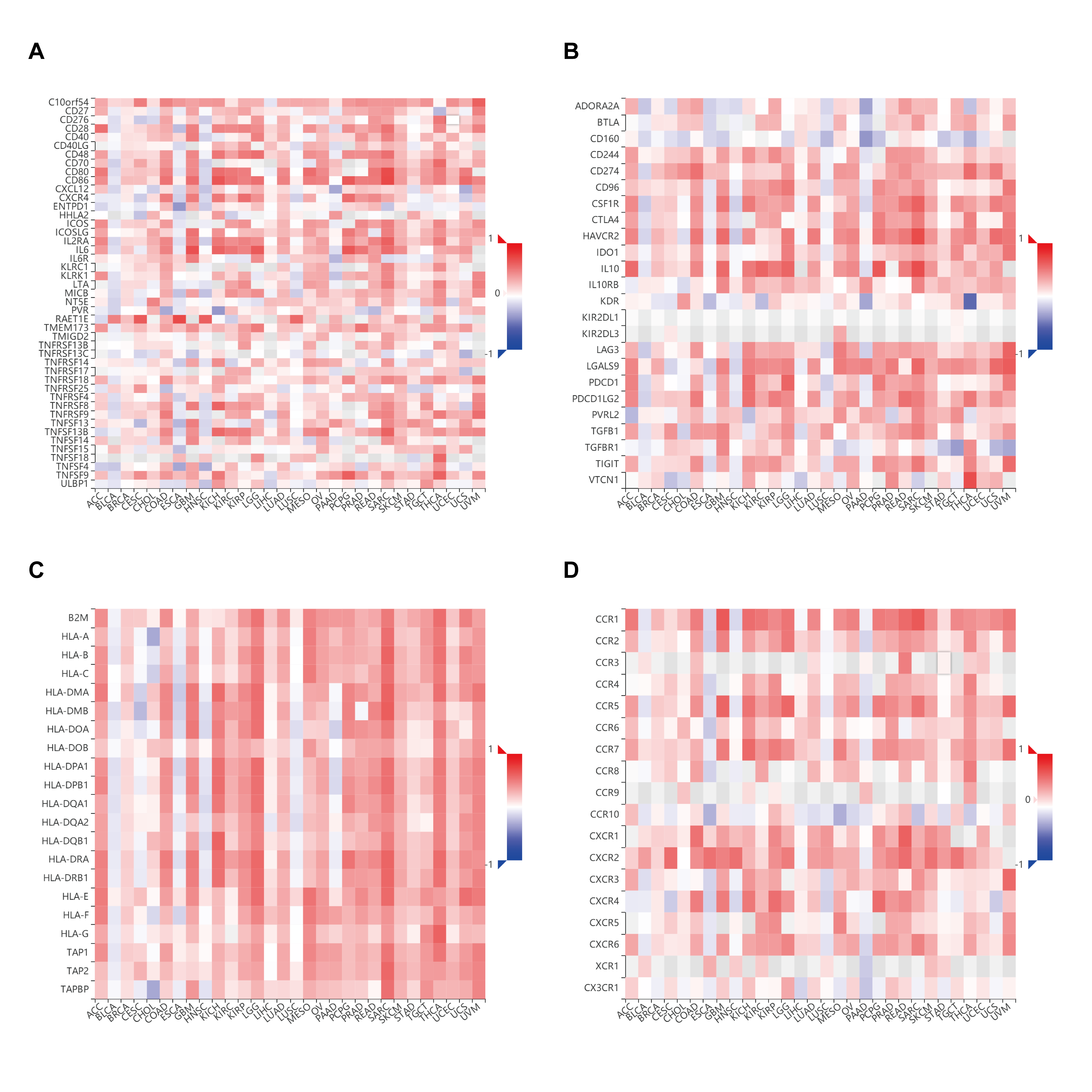

Supplement: Supplementary file 1 — Fig. S1. IL1RN expression is closely related to immunity across human cancers. Correlation analysis between the expression of IL1RN and the levels of (A) immune activating cytokines, (B) immune suppressive cytokines, (C) MHCs, and (D) chemokine receptors. [file 12885_2020_7620_MOESM1_ESM.tif]

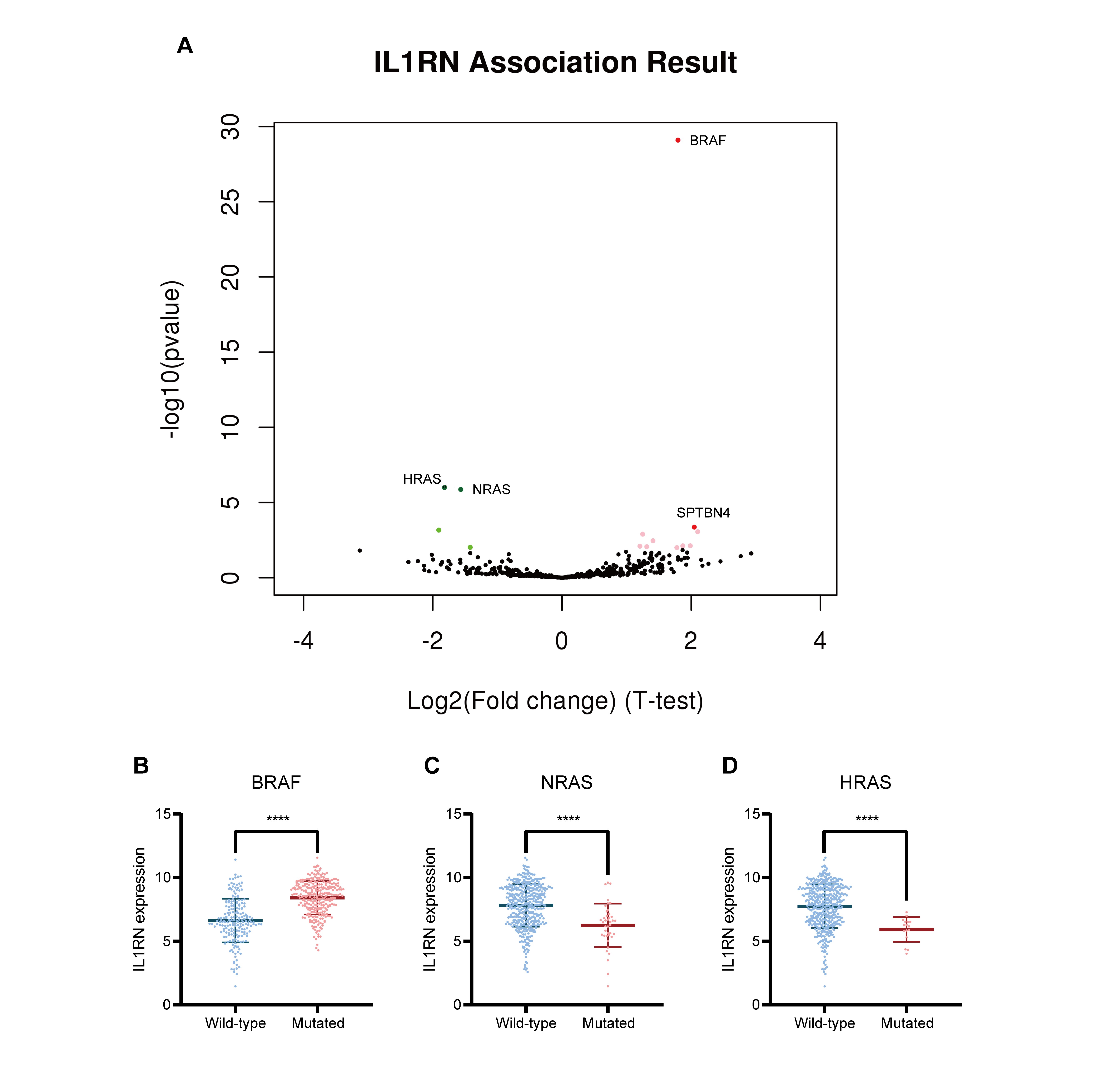

Supplement: Supplementary file 2 — Fig. S2. Relationship between IL1RN expression and gene mutations in PTC. (A) Volcano plot of IL1RN expression and gene mutations in THCA. Relationship between IL1RN expression and (B) BRAF mutation, (C) NRAS mutation, and (D) HRAS mutation. [file 12885_2020_7620_MOESM2_ESM.tif]
